# Isolation, Identification, and Antibiotic Resistance, CRISPR System Analysis of *Escherichia coli* from Forest Musk Deer in Western China

**DOI:** 10.3390/microorganisms13071683

**Published:** 2025-07-17

**Authors:** Kaiwei Yang, Xi Wu, Hui Ding, Bingcun Ma, Zengting Li, Yin Wang, Zexiao Yang, Xueping Yao, Yan Luo

**Affiliations:** 1 College of Veterinary Medicine, Sichuan Agricultural University, Chengdu 611130, China; 2023203025@stu.sicau.edu.cn (K.Y.); 2022103017@stu.sicau.edu.cn (X.W.); 2022303115@stu.sicau.edu.cn (H.D.); 10334@sicau.edu.cn (Y.W.); 13643@sicau.edu.cn (Z.Y.); 13577@sicau.edu.cn (X.Y.); 2 Sichuan Institute for Drug Control, Chengdu 611731, China; mbc1234@126.com (B.M.); cicilyok@126.com (Z.L.)

**Keywords:** forest musk deer, *Escherichia coli*, resistance genes, antibiotic resistance, CRISPR-Cas system

## Abstract

*Escherichia coli* (*E. coli*) is an opportunistic pathogen widely distributed in nature, and multi-drug resistance (MDR) *E. coli* has been widely recognized as a critical reservoir of resistance genes, posing severe health threats to humans and animals. A total of 288 *E. coli* strains were isolated and purified from fresh fecal samples of forest musk deer collected from farms in Sichuan, Shaanxi, and Yunnan Provinces of China between 2013 and 2023. This study aimed to conduct antibiotic susceptibility testing and resistance gene detection on the isolated forest musk deer-derived *E. coli*, analyze the correlations between them, investigate the presence of CRISPR systems within the strains, and perform bioinformatics analysis on the CRISPR systems carried by the strains. Results showed that 138 out of 288 *E. coli* strains were MDR, with the highest resistance to tetracycline (48.3%), cefalexin (45.1%), and doxycycline (41.7%). Prevalent genes were *tetA* (41.0%), *sul2* (30.2%), *blaTEM* (27.1%), with 29 gene–phenotype pairs correlated. CRISPR system-negative strains had higher resistance rates to 16 antibiotics and lower detection rates only for *aac (6′)-Ib-cr*, *qnrA*, and *qnrB* compared to CRISPR system-positive strains. Regional analysis showed that the problem of drug resistance in Sichuan and Shaanxi was more serious, and that the detection rate of antibiotic resistance genes was relatively high. This study guides *E. coli* infection control in forest musk deer and enriches resistance research data.

## 1. Introduction

Antibiotic resistance has emerged as a global crisis and a pressing health threat that demands urgent resolution. Antimicrobial resistance (AMR) has made the treatment of animal diseases more difficult, increasing the morbidity and mortality of animals, and severely affecting the production efficiency of animal husbandry. After wild animals come into contact with multidrug-resistant bacteria in the environment, they may further enhance the transmission of multidrug-resistant bacteria in nature, thereby affecting the health and stability of the entire ecosystem [1,2]. With the proposal of the “One Health” concept, increasing attention is being paid to the threat of animal-derived bacteria to human health [3]. Human exposure to animal-derived drug-resistant bacteria through air, food, and water sources increases the risk of antibiotic treatment failure [4].

As a major member of the intestinal microbiota in humans and animals, *E. coli* is a widely distributed Gram-negative bacterium and an important zoonotic conditional pathogen. Due to its strong survival ability and extreme tolerance to temperature and acidity, it easily infects humans and animals through water, soil, and food, causing severe diarrhea, septicemia, and various inflammatory diseases such as peritonitis, pneumonia, pericarditis, and cellulitis. *E. coli* infections not only pose a serious threat to the health of livestock and poultry in aquaculture but also cause significant economic losses [5]. Antibiotics are the primary method for treating *E. coli* diseases, but the continuous development of intensive and large-scale farming models has led to the widespread abuse of antibiotics worldwide, resulting in enhanced bacterial resistance and an increase in the number of superbugs [6]. As a typical multi-drug resistance bacterium, some bacterial multidrug resistance (MDR) *E. coli* strains carrying multiple resistance genes may even render commonly used antibiotics and so-called “last-resort” antibiotics ineffective.

MDR can be acquired through multiple pathways, such as mutations in antibiotic-targeted genes and the transfer and recombination of resistance genes [7]. The horizontal transfer of resistance genes is a crucial factor in the rapid spread of bacterial resistance. As a major reservoir of antibiotic resistance genes, *E. coli* can both acquire resistance genes from other bacteria and transfer its own resistance genes to other bacteria [8]. Antibiotic resistance genes (ARGs) are considered the main factor contributing to bacterial resistance and have been defined as new environmental pollutants. More than half of the total global antibiotic consumption is used in animal husbandry, thereby establishing the livestock industry as a primary source of resistance genes. High detection rates of resistance genes have been reported in wastewater, soil, and other environments around farms worldwide. Once these resistance genes spread to the surrounding environment, they augment the probability of human exposure. Therefore, strengthening investigations into bacterial resistance in animal farms and understanding the abundance of resistance genes are of utmost importance [9].

The Clustered Regularly Interspaced Short Palindromic Repeats-associated protein (CRISPR-Cas) system is a bacterial immune system composed of clustered, regularly interspaced short palindromic repeats and multiple CRISPR-associated genes. The CRISPR-Cas system is harbored by approximately 40% of bacteria and 90% of archaea [10]. It is the only adaptive immune system found in prokaryotes, consisting of two main parts: the CRISPR locus and Cas genes. The CRISPR locus includes a leader sequence, spacers, and direct repeats (DR). The CRISPR-Cas system is categorized into two classes based on the structural and functional characteristics of Cas proteins and further subcategorized into six types (I-VI) [11]. Among them, the Type II system is widely used in genome editing because it only requires one Cas protein to function. Current studies have demonstrated that an intact CRISPR system can prevent bacteria from acquiring plasmids carrying resistance genes, thereby reducing bacterial tolerance to antibiotics [12]. The absence of Cas genes in the CRISPR system may be one of the causes of multi-drug resistance bacteria [13,14].

The forest musk deer (*Moschus berezovskii*) is a mammal belonging to the order *Artiodactyla*, family *Moschidae*, and genus *Moschus*. In China, forest musk deer are mainly distributed in Sichuan, Shaanxi, Gansu, Yunnan, and other regions. The abdominal scent gland of adult male musk deer secretes musk, which is an essential raw material for high-end cosmetics and perfumes and a precious medicinal herb in traditional Chinese medicine [15]. Due to overhunting by humans, wild forest musk deer resources have been depleted, and the species has been listed as a national first-class protected wild animal in China and included in the International Union for Conservation of Nature (IUCN) Red List of Endangered Species in 2015. With the increasing severity of bacterial resistance, the incidence of bacterial diseases in captive forest musk deer has become higher and more harmful, posing a significant threat to the health of captive forest musk deer [16,17].

Currently, there is insufficient research on the resistance of forest musk deer-derived *E. coli*. Thus, this study aimed to characterize the antibiotic resistance phenotypes and antibiotic resistance genes profiles of *E. coli* isolates from Sichuan, Shaanxi, and Yunnan Provinces, China. We also conducted bioinformatics analysis of their CRISPR systems to elucidate the interconnections among resistance phenotypes, resistance genes, and CRISPR systems in forest musk deer-derived *E. coli*, thereby providing theoretical foundations for *E. coli* prevention and control.

## 2. Materials and Methods

### 2.1. Test Strains

The 288 *E. coli* strains used in this study were isolated from 315 fresh fecal samples randomly collected from forest musk deer farms in Sichuan, Shaanxi, and Yunnan Provinces between 2013 and 2023. Samples were stored in sterile centrifuge tubes (Eppendorf, Germany) and transported back to the laboratory at 4 °C within 12 h for bacterial isolation. Fecal samples were pre-enriched in LB broth medium (Haibo, Qingdao, China) at 37 °C with shaking at 120 r/min for 8 h. The culture was then spread onto EMB agar medium (Haibo, Qingdao, China) and incubated at 37 °C for 16 h. Single colonies exhibiting green metallic sheen on EMB agar were picked for Gram staining, and suspected *E. coli* colonies were purified and subjected to further identification testing.

### 2.2. DNA Extraction and 16S rRNA Gene Analysis

Bacterial DNA was extracted using a bacterial genomic DNA extraction kit (Tiangen, Beijing, China) according to the manufacturer’s instructions. The universal primers for the bacterial 16S rRNA gene (27F: 5′-AGAGTTTGATCCTGGCTCAG-3′ and 1492R: 5′-GGTTACCTTGTTACGACTT-3′) were used to amplify the 16S rRNA gene of the purified isolates [18]. The PCR reaction system (25 μL) comprised 12.5 μL of 2 × San Taq PCR Mix (Sangon, Shanghai, China), 1 μL each of forward and reverse primers (10 μmol/L), 1 μL of bacterial DNA, and 9.5 μL of ddH2O. The PCR amplification program was as follows: pre-denaturation at 94 °C for 2 min; denaturation at 94 °C for 30 s, primer annealing for 30 s, extension at 72 °C for 1 min, for a total of 30 cycles; and final extension at 72 °C for 10 min. The amplified products were submitted to Qingke Biological Co., Ltd. (Beijing, China) for sequencing, and the sequencing results were subjected to BLAST comparison in NCBI version 2.16.0. Colonies confirmed as *E. coli* after sequence alignment were purified on EMB agar medium.

### 2.3. Antibiotic Susceptibility Testing

In combination with a survey of antibiotic usage in each farm and the guidelines and interpretive standards of the Clinical and Laboratory Standards Institute (CLSI), the Kirby-Bauer (K-B) disk diffusion method was used to assess the sensitivity of the isolated bacteria to 24 antibiotics [19]. The antibiotics tested included piperacillin–tazobactam 10:1 (TZP, 100/10 μg), ampicillin–sulbactam 1:1 (SAM, 20/10 μg), amoxicillin–clavulanic acid 2:1 (AMC, 20/10 μg), cefalexin (CFX, 30 μg), ceftazidime (CAZ, 30 μg), cefuroxime (CXM, 30 μg), ceftriaxone (CRO, 30 μg), cefazolin (KZ, 30 μg), cefoxitin (FOX, 30 μg), aztreonam (ATM, 30 μg), imipenem (IPM, 10 μg), meropenem (MEM, 10 μg), gentamicin (CN, 10 μg), tobramycin (TOB, 10 μg), amikacin (AK, 30 μg), trimethoprim–sulfamethoxazole (STX, 25 μg), polymyxin B (PB, 300 μg), nitrofurantoin (F, 100 μg), levofloxacin (LEV, 5 μg), ciprofloxacin (CIP, 5 μg), florfenicol (FFC, 30 μg), chloramphenicol (CHL, 30 μg), tetracycline (TE, 30 μg), and doxycycline (DOX, 5 μg). Results were interpreted according to CLSI standards. *E. coli* ATCC25922 was used as a control [20]. MDR strains were defined as those resistant to three or more classes of antibiotics [21].

### 2.4. Antibiotic Resistance Gene Detection

PCR detection methods were established for representative antibiotic resistance genes (ARGs) in *E. coli* strains, including β-lactams (*blaTEM*, *blaCTX-M*, *blaSHV*), tetracyclines (*tetA*, *tetX*), quinolones (*qnrA*, *qnrB*, *qnrS*), amide alcohols (*floR*), sulfonamides (*sul1*, *sul2*, *sul3*), and aminoglycosides (*aac(6′)-Ib-cr*, *strA*, *strB*, *gyrB*, *ant(3″)-Ia*, *aph(3′)-Ⅱa*) resistance genes. The PCR primers and corresponding annealing temperatures are shown in Table S1 [22].

### 2.5. CRISPR Sequence Analysis

The presence of the CRISPR system in the isolated strains was detected by PCR. Primers were designed based on the CRISPR structure sequences of *E. coli* published on the CRISPR database website (https://crisprcas.i2bc.paris-saclay.fr/, accessed on 3 March 2025.) (CF: 5′-TACCGTTGGTGAAGGAGCTG-3′, CR: 5′-TTCCGGTGGATTTGGATGGG-3′). The repeat and spacer sequences of the amplified CRISPR sequences were analyzed using the CRISPRCasFinder website (https://crisprcas.i2bc.paris-saclay.fr/, accessed on 5 March 2025) to identify potential CRISPR targets. The identified spacer sequences of each strain were submitted to the CRISPRTarget website (https://crispr.otago.ac.nz/CRISPRTarget/crispr_analysis.html, accessed on 7 March 2025) for BLAST comparison analysis of the spacer sequence sources against the GenBank-Phage, RefSeq-Plasmid, RefMicrobial, and RefSeq-viral databases (using default parameters), with sequences showing that > 90% were considered homologous. The RNA secondary structure of the repeat sequences was predicted using RNAfold web (http://rna.tbi.univie.ac.at/cgi-bin/RNAWebSuite/RNAcofold.cgi, accessed on 10 March 2025).

### 2.6. Data Processing and Analysis

Statistical analysis was performed using SPSS Statistics Version 26. *p*-values < 0.05 were considered statistically significant. The Pearson correlation coefficient (r) was used as a nonparametric measure of the strength of association between two variables, ranging from −1 to 1 [23]. The assessment of correlation was conducted using the chi-square test and by computing Spearman’s rank correlation coefficient (r). Specifically, according to the previous literature, an absolute value of r in the range of 0.5 to 1.0 denoted a high correlation, 0.3 to 0.5 indicated a moderate correlation, 0.1 to 0.3 signified a low correlation, and an absolute value of r < 0.1 indicated no correlation [5].

## 3. Results

### 3.1. Antibiotic Resistance Phenotype Analysis

A total of 288 strains of *Escherichia coli* strains were isolated in this study, including 163 strains from Sichuan Province, 70 strains from Shaanxi Province, and 55 strains from Yunnan Province in China. Among all 288 isolated *E. coli* strains, 138 (47.92%, 138/288) were resistant to three or more classes of antibiotics and defined as MDR *E. coli*. Specifically, MDR *E. coli* isolates from Sichuan, Shaanxi, and Yunnan were 81 (49.69%, 81/163), 40 (57.14%, 40/70), and 17 (30.91%, 17/55), respectively. Furthermore, 51 (17.70%, 51/288) *E. coli* strains were sensitive to all antibiotics. As shown in Table 1, among the eight classes of antibiotics used in the susceptibility test, β-lactam antibiotics had the highest resistance rate (73.61%, 212/288), followed by tetracycline antibiotics (50.35%, 145/288), aminoglycoside antibiotics (41.32%, 119/288), sulfonamide antibiotics (36.11%, 104/288), amide alcohol antibiotics (32.29%, 93/288), quinolone antibiotics (28.47%, 82/288), furan antibiotics (2.43%, 7/288), and polypeptide antibiotics (1.74%, 5/288). Among the 24 antibiotics used, tetracycline (48.26%, 139/288), cefalexin (45.14%, 130/288), and doxycycline (41.67%, 120/288) had the highest resistance rates, while cefoxitin (3.13%, 9/288), nitrofurantoin (2.43%, 7/288), and polymyxin B (1.74%, 5/288) had the lowest resistance rates, with resistance rates of other antibiotics ranging between 4.17% and 37.5%. As shown in Table S2 the detailed analysis of resistance phenotypes revealed 194 distinct resistance profiles among the 288 *E. coli* isolates, with IPM (5.90%, 17/288), TET/DOX (4.51%, 13/288), AK/IPM (1.74%, 5/288), CFX (1.74%, 5/288), and CHL/AMC/CFX/CAZ/CXM/CRO/KZ/ATM (1.39%, 4/288) being the most common.

Table 2 details the resistance rates of *E. coli* strains isolated from Sichuan, Shanxi, and Yunnan Provinces to 24 antibiotics. Among them, 163 *E. coli* strains isolated from Sichuan had higher resistance rates to 19 antibiotics than the overall average across the three regions, 70 strains isolated from Shaanxi had higher resistance rates to 15 antibiotics than the overall average, and 55 strains isolated from Yunnan had a higher resistance rate only to FOX than the overall average. Comparison of the resistance rates of the isolated strains from the three regions to various antibiotics showed that the resistance rates of AK, FFC, CIP, TZP, SAM, AMC, CFX, CAZ, CXM, CRO, KZ, ATM, IPM, and F were highest in Sichuan, while the resistance rates of CN, TOB, TET, DOX, CHL, LEV, SXT, MEM, and PB were highest in Shaanxi. The isolated strains from Yunnan had a higher resistance rate only to FOX than the other two regions and showed no resistance to AK, TZP, SAM, IPM, PB, and F.

### 3.2. Distribution of Resistance Genes in E. coli

As shown in Table 3, 276 *E. coli* strains were detected with at least one resistance gene. Among the 18 resistance genes across six classes tested, 16 resistance genes were detected, with *gyrB* (90.97%), *tetA* (40.97%), and *sul2* (30.21%) having the highest detection rates, while *tetX* (0.35%), *qnrA* (0.35%), and *aac(6′)-Ib-cr* (3.13%) had the lowest detection rates. The detection rates of the remaining 10 detected resistance genes ranged from 4.51% to 23.61%, and *aph (3′)-IIa* and *blaSHV* were not identified.

Table 4 shows the detection rates of resistance genes in the three regions. Sichuan had higher detection rates than the overall average across the three regions for seven resistance genes, including *ermB*, *sul3*, *qnrA*, *qnrB*, *aac(6′)-Ib-cr*, *ant (3′’)-Ia*, and *tetX*. Shaanxi had higher detection rates than the overall average for 10 resistance genes, including *floR*, *blaTEM*, *sul1*, *sul2*, *strA*, *gyrB*, *qnrS*, *strB*, *blaCTX-M*, and *tetA*. The isolated strains from Yunnan had lower detection rates of all resistance genes than the overall average and the other two regions. The detailed profiles of antibiotic resistance genes harbored by all isolated strains are provided in Supplementary Table S3.

### 3.3. Relationship Between ARGs and AMRs

**Figure 1:**
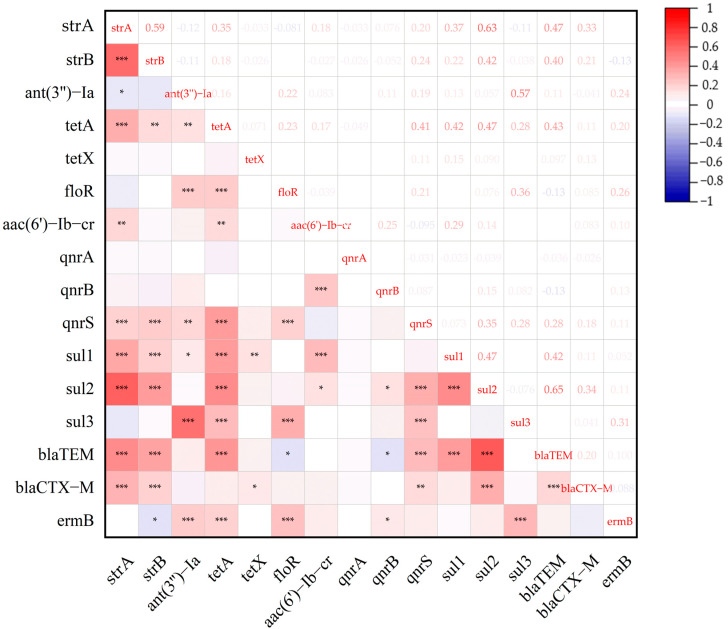
The associations between ARGs among *E. coli* isolates from musk deer (*n* = 288). The intensity of the color represents the value of the correlation coefficient (r). * *p* ≤ 0.05 ** *p* ≤ 0.01 *** *p* ≤ 0.001.

Figure 1 illustrates 48 pairs of positive correlations (r > 0.1, *p* < 0.05) among ARGs, with the strongest correlations found between *blaTEM* and *sul2* (r = 0.65), *sul2* and *strA* (r = 0.63), *strA* and *strB* (r = 0.59), and *sul3* and *ant(3′′)-Ia* (r = 0.57). The correlations among the remaining ARGs ranged from r = 0.12 to 0.47. Four pairs of negative correlations (r < −0.1, *p* < 0.05) were also observed among ARGs, including *floR* and *blaTEM* (r = −0.13), *blaTEM* and *qnrB* (r = −0.13), *strA* and *ant(3′′)-Ia* (r = −0.12), and *strB* and emrB (r = −0.13).

**Figure 2:**
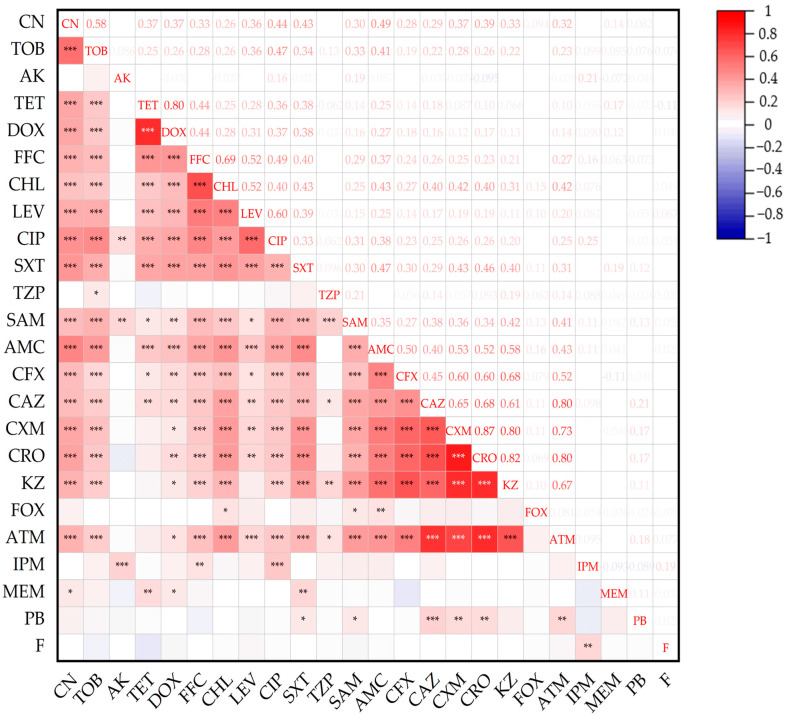
The associations between AMRs among *E. coli* isolates from musk deer (*n* = 288). The intensity of the color represents the value of the correlation coefficient (r). * *p* ≤ 0.05 ** *p* ≤ 0.01 *** *p* ≤ 0.001.

As shown in Figure 2, there were 155 pairs of positive correlations (r > 0.1, *p* < 0.05) among AMRs, with the strongest positive correlations observed in five pairs: CXM and CRO (r = 0.87), CRO and KZ (r = 0.82), CRO and ATM (r = 0.81), CXM and KZ (r = 0.80), and TE and DOX (r = 0.80). Additionally, 18 pairs showed strong positive correlations (r = 0.51–0.79), and the remaining 93 pairs had positive correlations ranging from r = 0.21 to 0.50.

**Figure 3:**
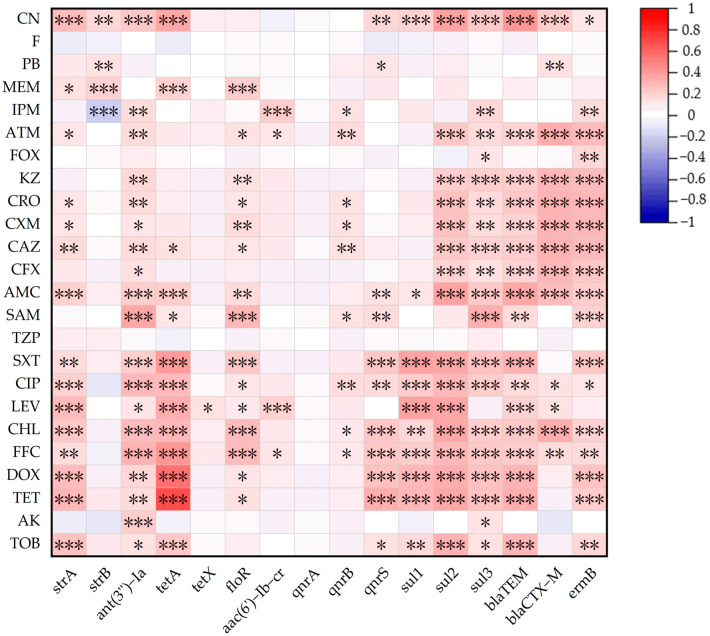
The associations between AMRs and ARGs among *E. coli* isolates from musk deer (*n* = 288). The intensity of the color represents the value of the correlation coefficient (r). * *p* ≤ 0.05 ** *p* ≤ 0.01 *** *p* ≤ 0.001.

As shown in Figure 3, there were 184 pairs of positive correlations (r > 0.1, *p* < 0.05) between ARGs and AMRs. Among them, 29 ARG–AMR pairs of resistance genes matched their mediated antibiotic resistance phenotypes. The *tetA* gene showed the strongest correlations with tetracycline antibiotics TE and DOX (r = 0.693, r = 0.572). For aminoglycoside antibiotics, CN was positively correlated with *strA*, *strB*, and *ant(3′′)-Ia* (r = 0.295, r = 0.181, r = 0.232), TOB was positively correlated with *strA* and *ant(3′′)-Ia* (r = 0.263, r = 0.163), and AK was positively correlated with *ant(3′′)-Ia* (0.229). For amide alcohol antibiotics, both FFC and CHL showed positive correlations with *floR* (r = 0.279, r = 0.302). For quinolone antibiotics, LEV was positively correlated with *aac(6′)-Ib-cr* (r = 0.216), and CIP was positively correlated with *qnrB* and *qnrS* (r = 0.162, r = 0.173). For sulfonamide antibiotics, STX was positively correlated with *sul1*, *sul2*, and *sul3* (r = 0.378, r = 0.366, r = 0.281). For β-lactam antibiotics, *blaTEM* was positively correlated with AMC, CFX, CAZ, CXM, CRO, KZ, and ATM (r = 0.366, r = 0.217, r = 0.221, r = 0.211, r = 0.243, r = 0.219, r = 0.200), and *blaCTX-M* was positively correlated with AMC, CFX, CAZ, CXM, CRO, KZ, and ATM (r = 0.282, r = 0.317, r = 0.325, r = 0.322, r = 0.322, r = 0.303, r = 0.353).

### 3.4. Characteristics Analysis of CRISPR System in Isolated Strains

**Figure 4:**
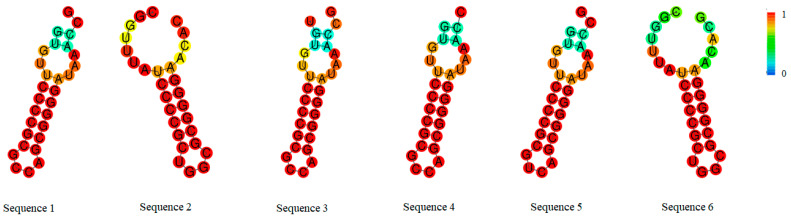
Prediction of secondary structure of *E. coli* repeat sequences. The structure is colored by base-pairing probabilities.

Among the 288 *E. coli* isolates, 131 strains were detected with CRISPR structures, with a detection rate of 45.49%. A total of six types of repeat sequences were identified in the detected CRISPR structures. The repeat sequences in the CRISPR structure can be transcribed into RNA and form stem-loop secondary structures, which can serve as targets for CAS protein recognition and further cleavage [24]. As shown in Figure 4, the secondary structures of the six repeat sequences identified in this study were predicted. Based on the stem-loop structures, they can be divided into two categories. Sequences 1, 3, 4, and 5 had two loop structures in the head and middle regions, with the head loop comprising six bases, the middle loop nine bases, and the inter-loop stem region measuring five bases in length. The differences between sequences 1, 3, 4, and 5 mainly lie in the tail, with sequences 1 and 5 having two bases at the tail, and sequences 3 and 4 having three bases and one base at the tail, respectively. Sequences 2 and 6 had only one loop structure composed of six bases in the head in their secondary structures, and compared to other sequences, sequences 2 and 6 had longer tail sequences, reaching thirteen bases. As shown in Table 5, the minimum free energy of repeat sequences 1, 3, 4, and 5 was −15.20 kcal/mol, while that of sequences 2 and 6 was −14.20 kcal/mol.

The spacer sequences found in *E. coli* were compared with sequences in four databases (GenBank-Phage, RefSeqPlasmid, RefSeq-Microbial, and RefSeq-viral) using BLAST. A total of 1624 spacer sequences were identified using CRISPR Finder software version 1.1.2, and after excluding duplicate spacer sequences, 375 unique spacer sequences were counted. The CRISPR Target software version 6.1 was used to compare the sources of the identified non-duplicate spacer sequences, and 243 spacer sequences were found to have homologous fragments in the databases, including 148 from plasmids, 91 from phages, and 4 from viruses.

**Figure 5:**
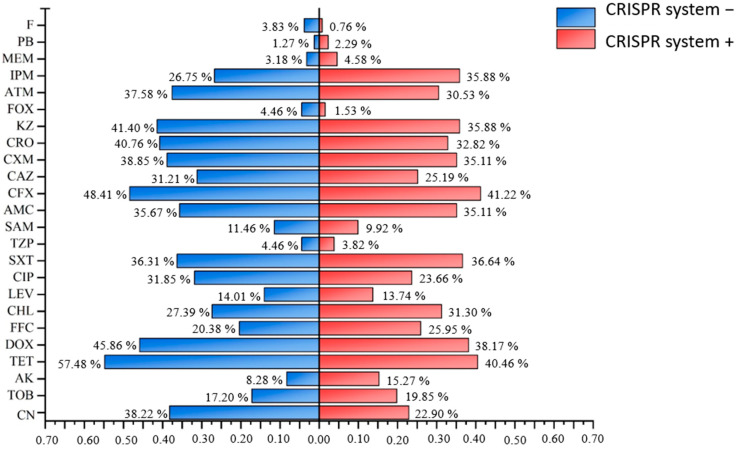
Differences in resistance rates to various antibiotics between strains carrying and not carrying CRISPR systems. The antibiotic resistance rates of strains with and without the CRISPR system are, respectively, represented by red and blue.

**Figure 6:**
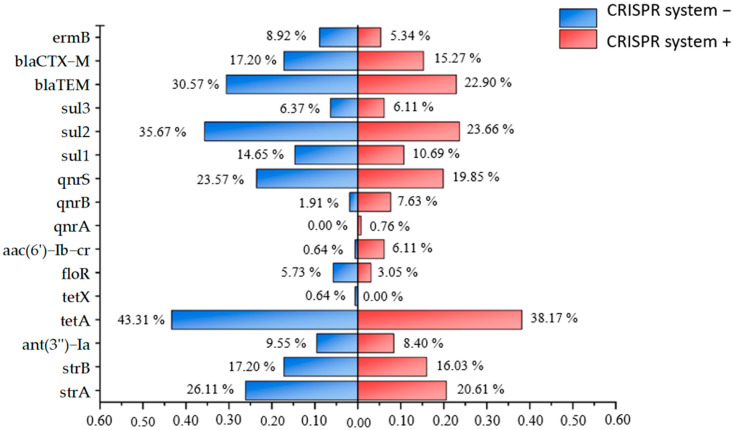
Differences in carrying rate of antibiotic resistance genes between strains carrying and not carrying CRISPR systems. The carriage rates of antibiotic resistance genes in strains with and without the CRISPR system are, respectively, represented by red and blue.

The comparison of antibiotic resistance rates and resistance gene detection rates between CRISPR system-positive and -negative strains, as shown in Figure 5 and Figure 6, showed that CRISPR system-negative strains had higher resistance rates to 16 antibiotics, including TET, DOX, LEV, CIP, TZP, SAM, AMC, CFX, CAZ, CXM, CRO, KZ, FOX, ATM, and F, compared to CRISPR system-positive strains. Among the 16 resistance genes detected in this experiment, CRISPR system-negative strains had lower detection rates only for *aac(6′)-Ib-cr*, *qnrA*, and *qnrB* compared to CRISPR system-positive strains. Additionally, 39.69% of CRISPR system-positive strains were MDR, while 54.78% of CRISPR system-negative strains were MDR. These results suggest that the CRISPR system may interfere with the acquisition of resistance genes and the development of resistance in *E. coli* isolates to a certain extent.

## 4. Discussion

Currently, antibiotic therapy remains the primary strategy for controlling bacterial infections globally [25]. However, the prevalence of MDR *E. coli* has been increasing, and with the rise in antibiotic resistance and the slow development of new antibiotics, *E. coli*-induced infections are imposing a growing health burden and mortality risk [26,27]. Antimicrobial susceptibility testing is crucial for guiding the selection of appropriate antimicrobial agents to treat infections and control the prevalence of MDR bacteria.

According to the resistance profiles of the isolated strains in this study, 288 *E. coli* strains exhibited 193 resistance phenotypes. The complex resistance phenotypes of the isolated strains reflect the rapid and diverse changes in bacterial resistance patterns, which undoubtedly pose challenges to the treatment of bacterial infections in forest musk deer. Monitoring the resistance of strains isolated from farms, summarizing their resistance phenotypes, and using this information to guide the prudent and rational use of antibiotics will help select more effective prevention and treatment strategies in forest musk deer farming.

The highest resistance rate observed in this study was for tetracycline, which is consistent with the findings of several studies by Tao He [28], Katarzyna Ćwiek [29], Hanaa M El Maghraby [30], and others. This trend is not surprising, as tetracycline-class drugs are among the most commonly used antibiotics in aquaculture in China and globally. Their extensive use may contribute to the development of resistance in *E. coli* [31,32,33]. The high prevalence of antibiotic resistance among the 288 *E. coli* isolates partially reflects the issue of irrational antibiotic use in forest musk deer farms, highlighting the urgency of rational selection of antimicrobials in forest musk deer farming. However, the isolated strains in this study showed significantly lower resistance rates to meropenem, piperacillin–tazobactam, cefoxitin, polymyxin B, and nitrofurantoin, all below 10%, with PB having the lowest resistance rate at 1.74%. Based on the results of this experiment, the use of tetracyclines, quinolones, and aminoglycosides should be restricted in subsequent forest musk deer farming, while polypeptide antibiotics should be considered as a priority for treating *E. coli* infections in forest musk deer. Polypeptide antibiotics exhibit broad-spectrum activity against Gram-positive/negative bacteria, fungi, viruses, and protozoa. Unlike conventional antibiotics, they act by disrupting cell membranes or targeting multiple pathways, posing lower resistance risks [34]. Additionally, polypeptide antibiotics are poorly absorbed in the animal intestine, acting primarily in the digestive tract, with extremely low residual levels in the body, ensuring high safety of use. As natural or synthetic antimicrobial molecules composed of amino acids, polypeptide antibiotics have become important alternatives to traditional antibiotics in recent years, demonstrating significant advantages in growth promotion and disease treatment in animal farming [35].

Analysis of the resistance gene carriage rates in different regions showed that Yunnan had the lowest carriage rates of all resistance genes, significantly lower than the average of the three regions, while Shaanxi had higher detection rates of 10 resistance genes than the other two regions, with *sul2*, *strA*, *strB*, and *blaTEM* showing much higher detection rates than Sichuan and Yunnan. The differences in antibiotic susceptibility test results and resistance gene detection rates among the three regions of Sichuan, Shaanxi, and Yunnan may be primarily attributed to differences in the types, frequency, and intensity of antibiotic use in each region, as evidenced by the findings of Nicola M Pfeifer [36] and others on the impact of antibiotic selection pressure on resistance phenotypes. Secondly, environmental and ecological factors may also indirectly influence the spread of bacterial resistance. Studies have shown that differences in climate zones can indirectly regulate the transmission dynamics of resistance genes by affecting the structure of environmental microorganisms. For example, in warmer environments, the proportion of strains carrying carbapenem and β-lactam resistance genes increases significantly [37]. Environmental media such as soil and water in different regions of ecosystems also influence the spread of resistance genes and the colonization of resistant strains [38]. Furthermore, the genetic background of strains and adaptive evolution may also contribute to these phenomena [39].

Notably, a *tetX*-carrying *E. coli* strain was isolated from Sichuan in this experiment. This gene can cause high-level resistance to tigecycline, which is an irreplaceable treatment for MDR bacterial infections in humans. With the increasing severity of carbapenem antibiotic resistance in recent years, tigecycline has become the optimal choice for treating MDR bacterial infections and has been designated by the World Health Organization (WHO) as the last line of defense against infections caused by multi-drug resistance Gram-negative bacteria, classified as a critical antimicrobial agent requiring strict restriction of use. Tigecycline has a mechanism of action similar to other tetracyclines, inhibiting bacterial protein synthesis by binding to the 30S subunit of the ribosome [40,41]. Although tigecycline is not permitted for use in animal husbandry, numerous cases of plasmid-mediated tigecycline resistance gene *tetX* have been detected in animal-derived bacteria in recent years, increasing the probability of horizontal transmission of *tetX*. If this leads to clinical treatment failure in humans, it could potentially trigger a global public health crisis. Some researchers speculate that the increased detection rate of *tetX* in animal-derived bacteria in recent years may be related to the long-term selection pressure caused by the extensive use of tetracycline-class antibiotics in regions such as China, the United States, and Europe over recent decades, which may have promoted the emergence and spread of *tetX* [42]. Mobile plasmids such as IncQ, IncX1, and IncFIB play a crucial role in this process. Studies have found that *tetX*-positive plasmids with the same Inc type were detected in *E. coli* strains with the same or different ST types, further confirming the important role of plasmids in the transmission of the *tetX* gene. Additionally, the mobile element ISCR2 has been confirmed to mediate the transmission of *tetX* between plasmids and chromosomes in different hosts, and ISCR2 is present in the upstream or downstream genes of multiple *tetX* mutants such as *tet(X3)*, *tet(X4)*, and *tet(X5)* [43,44,45,46]. Therefore, monitoring the prevalence of *tetX* in animal-derived *E. coli* has become increasingly urgent.

Analysis of the correlation between ARGs and AMRs in the isolated strains revealed 191 pairs of positive correlations. However, there were also phenomena where resistance phenotypes did not match resistance gene carriage, such as some isolated strains carrying only a few ARGs (e.g., *qnrA*, *tetA*, *sul2*, and *ermB*) or no ARGs at all but still showing resistance to some antibiotics, while other strains carried resistance genes but did not exhibit resistance to the corresponding antibiotics. Possible reasons for these phenomena include the following. (1) Bacteria exhibit different sensitivities to antibiotics when in different metabolic states. For example, bacteria in a non-dividing state with low metabolic activity show increased sensitivity to ciprofloxacin and streptomycin but reduced sensitivity to penicillin and tetracycline, a phenomenon known as “drug indifference”, which is common in chronic infections. When treatment is stopped, some bacteria may recover from a quiescent state to a normal state, leading to disease recurrence [47,48,49,50]. (2) Carried resistance genes may be in a silent state and unexpressed, defined as “silent antibiotic resistance genes”. Bacterial resistance genes are mostly acquired from the outside through horizontal transfer, so bacteria often silence these foreign genes through various mechanisms to reduce survival costs. The expression of resistance genes can be influenced by their carriage in different bacterial species and the nutritional status of the bacteria. Although silent resistance genes do not confer antibiotic resistance, they can still be transmitted among bacterial populations, posing a potential threat to antibiotic therapy [51]. (3) Bacteria without resistance genes can also develop antibiotic resistance through biofilm formation, reduced growth rate, metabolic changes, and stimulating the expression of efflux pumps through metabolic products [52].

Previous studies have demonstrated that the spacer sequences in the bacterial CRISPR system share high homology with exogenous genes such as plasmids and phages and certain bacterial endogenous genes [24]. Therefore, in this study, 375 non-duplicate *E. coli* spacer sequences were subjected to BLAST comparison against four databases: GenBank-Phage, RefSeq-Plasmid, RefSeq-Microbial, and RefSeq-viral. A total of 243 spacer sequences had homologous matches in the databases, including 99 sequences homologous to *E. coli* phages or plasmids, confirming that *E. coli* defends against phages and exogenous plasmids by integrating exogenous genes into its CRISPR structure. Homologous sequences were also identified with plasmids and phages of other bacterial species such as Salmonella, Enterococcus, Citrobacter braakii, Escherichia fergusonii, Klebsiella pneumoniae, and Shigella flexneri. This may be due to the close genetic relationship and similar living environments of *E. coli* with these bacteria, as well as the animal intestine providing a favorable environment for horizontal gene transfer between these bacteria [53]. Although many spacer sequences were repetitive, the repeated appearance of these spacer sequences in different or the same strains indicates that the CRISPR structure of bacteria is constantly inserting and deleting spacer sequences during the evolution of bacteria [54]. The combination of repeat sequences and spacer sequences from different bacteria exhibits significant differences in arrangement and sequence, which may provide greater help for typing and tracing bacteria carrying CRISPR structures in the future [55,56].

It should be noted that this study had limitations, including the limited number of antibiotics and resistance genes tested, as well as transposons, insertion sequences, and integrons that were not considered. In future studies, facing the complex problem of bacterial resistance, whole-genome sequencing (WGS) technology should be used to provide a more comprehensive investigation of resistance gene carriage in isolated strains, combined with environmental conditions, genetic interactions, and bacterial adaptive mechanisms for a more comprehensive integrated analysis based on gene detection and resistance phenotype experiments.

## 5. Conclusions

In summary, this study isolated 288 *E. coli* strains from feces of captive forest musk deer in Sichuan, Shaanxi, and Yunnan Provinces of China, conducted antibiotic susceptibility testing, resistance gene detection, and CRISPR system carriage analysis, and analyzed the correlations among them. In terms of resistance, the isolated strains from the three regions showed high resistance rates to tetracycline, trimethoprim–sulfamethoxazole, and cefalexin, but Yunnan had relatively lower resistance rates to various antibiotics compared to the other two regions. For resistance genes, *tetA*, *strA*, *sul3*, and *blaTEM* had high detection rates, and correlation analysis revealed 29 ARG–AMR pairs with positive correlations between resistance genes and their mediated phenotypes. The proportions of MDR bacteria, antibiotic resistance rates, and resistance gene detection rates were compared between CRISPR system-positive and CRISPR system-negative strains. The results of this study provide a theoretical basis for the rational use of antibiotics in forest musk deer farms and the efficient prevention and treatment of bacterial diseases in forest musk deer. From the “One Health” perspective, conducting antibiotic susceptibility testing and resistance gene detection on animal-derived *E. coli* will also contribute to the common development of animal welfare, agricultural biosafety, and public health.

## Figures and Tables

**Table 1:** Antimicrobial resistance (AMR) detected in *E. coli* strains isolated from musk deer (*n* = 288).

Category of Antimicrobial	No. of Resistant Isolates (%)	Antibiotic	No. of Resistant Isolates (%)
aminoglycosides	119 (41.32)	gentamicin (CN)	90 (31.25)
		tobramycin (TOB)	53 (18.40)
		amikacin (AK)	33 (11.46)
tetracyclines	145 (50.34)	tetracycline (TET)	139 (48.26)
		doxycycline (DOX)	120 (41.67)
amide alcohols	93 (32.29)	florfenicol (FFC)	66 (22.92)
		chloramphenicol (CHL)	84 (29.17)
quinolones	82 (28.47)	Levofloxacin (LEV)	40 (13.89)
		ciprofloxacin (CIP)	51 (17.71)
sulfonamides	105 (36.11)	trimethoprim–sulfamethoxazole (SXT)	105 (36.46)
β-lactams	212 (73.61)	piperacillin/tazobactam10:1 (TZP)	12 (4.17)
		ampicillin/sulbactam 1:1 (SAM)	31 (10.76)
		amoxicillin/clavulanic acid 2:1 (AMC)	101 (35.07)
		cefalexin (CFX)	130 (45.14)
		ceftazidime (CAZ)	82 (28.47)
		cefuroxime sodium (CXM)	108 (37.5)
		ceftriaxone (CRO)	107 (37.15)
		cephazolin (KZ)	108 (37.5)
		cefoxitin (FOX)	9 (3.13)
		aztreonam (ATM)	99 (34.37)
		imipenem (IPM)	89 (30.9)
		meropenem (MEM)	15 (5.21)
polypeptides	5 (1.74)	polymyxin b (PB)	5 (1.74)
furans	7 (2.43)	nitrofurantoin (F)	7 (2.43)

**Table 2:** The distribution among *E. coli* resistance phenotype from musk deer in Sichuan (*n* = 163), Shanxi (*n* = 70), and Yunnan (*n* = 55).

Antibiotic	No. of Resistant Isolates in Sichuan (%)	No. of Resistant Isolates in Shanxi (%)	No. of Resistant Isolates in Yunnan (%)
Gentamicin (CN)	45 (27.61)	30 (42.86)	15 (27.27)
Tobramycin (TOB)	30 (18.40)	16 (22.86)	7 (12.73)
Amikacin (AK)	32 (19.63)	1 (1.43)	0 (0.00)
Tetracycline (TET)	79 (48.47)	40 (57.14)	20 (36.36)
Doxycycline (DOX)	73 (44.79)	32 (45.71)	15 (27.27)
Florfenicol (FFC)	43 (26.38)	16 (22.86)	7 (12.73)
Chloramphenicol (CHL)	49 (30.06)	28 (40.00)	7 (12.73)
Levofloxacin (LEV)	25 (15.34)	12 (17.14)	3 (5.45)
Ciprofloxacin (CIP)	35 (21.47)	13 (18.57)	3 (5.45)
trimethoprim–sulfamethoxazole (SXT)	60 (36.81)	26 (37.14)	19 (34.55)
piperacillin/tazobactam10:1 (TZP)	9 (5.52)	3 (4.29)	0 (0.00)
ampicillin/sulbactam 1:1 (SAM)	24 (14.72)	7 (10.00)	0 (0.00)
amoxicillin/clavulanic acid 2:1 (AMC)	62 (38.04)	24 (34.29)	15 (27.27)
cefalexin (CFX)	81 (49.69)	26 (37.14)	23 (41.82)
ceftazidime (CAZ)	57 (34.79)	21 (30.00)	4 (7.27)
cefuroxime sodium (CXM)	67 (41.10)	24 (34.29)	17 (30.91)
ceftriaxone (CRO)	66 (40.49)	26 (37.14)	15 (27.27)
cephazolin (KZ)	68 (41.72)	23 (32.86)	17 (30.91)
cefoxitin (FOX)	5 (3.07)	2 (2.86)	2 (3.64)
aztreonam (ATM)	68 (41.72)	25 (35.71)	6 (10.91)
imipenem (IPM)	86 (52.76)	3 (4.29)	0 (0.00)
meropenem (MEM)	7 (4.29)	7 (10.00)	1 (1.82)
polymyxin b (PB)	2 (1.23)	3 (4.29)	0 (0.00)
nitrofurantoin (F)	7 (4.29)	0 (0.00)	0 (0.00)

**Table 3:** Details of antimicrobial resistance (AMR) and antibiotic resistance genes (ARGs) detected in *E. coli* strains isolated from musk deer (*n* = 288).

Category of Antimicrobial	No. of Resistant Isolates (%)	ARGs	No. of Positive Isolates (%)
aminoglycosides	119 (41.32)	*strA*	68 (23.61%)
		*strB*	48 (16.67%)
		*gyrB*	262 (90.97%)
		*ant(3′′)-Ia*	26 (9.03%)
		*aph(3′)-IIa*	0 (0%)
tetracyclines	145 (50.34)	*tetA*	118 (40.97%)
		*tetX*	1 (0.35%)
amide alcohols	93 (32.29)	*flor* *ermB*	13 (4.51%) 21 (7.29%)
quinolones	82 (28.47)	*aac(6′)-Ib-cr*	9 (3.13%)
		*qnrA*	1 (0.35%)
		*qnrB*	13 (4.51%)
		*qnrS*	63 (21.88%)
sulfonamides	105 (36.11)	*sul1*	37 (12.85%)
		*sul2*	87 (30.21%)
		*sul3*	68 (23.61%)
β-lactams	212 (73.61)	*blaTEM*	78 (27.08%)
		*blaCTX-M*	47 (16.32%)
		*blaSHV*	0 (0%)

**Table 4:** The distribution of antibiotic resistance genes among *E. coli* from captive musk deer in Sichuan (*n* = 163), Shanxi (*n* = 70), and Yunan (*n* = 55).

ARGs	Number of Positive Strains in Sichuan (%)	Number of Positive Strains in Shanxi (%)	Number of Positive Strains in Yunnan (%)
*floR*	7 (4.29%)	6 (8.57%)	0 (0.00%)
*blaTEM*	40 (24.54%)	37 (52.86%)	1 (1.82%)
*sul1*	20 (12.27%)	16 (22.86%)	1 (1.82%)
*sul2*	46 (28.22%)	40 (57.14%)	1 (1.82%)
*strA*	24 (14.72%)	39 (55.71%)	5 (9.09%)
*gyrB*	148 (90.80%)	65 (92.86%)	49 (89.09%)
*qnrS*	29 (17.79%)	26 (37.14%)	8 (14.55%)
*strB*	8 (4.91%)	36 (51.43%)	4 (7.27%)
*blaCTX-M*	24 (14.72%)	18 (25.71%)	5 (9.09%)
*ermB*	19 (11.66%)	2 (2.86%)	0 (0.00%)
*sul3*	15 (9.20%)	1 (1.43%)	2 (3.64%)
*blaSHV*	0 (0.00%)	0 (0.00%)	0 (0.00%)
*qnrA*	1 (0.61%)	0 (0.00%)	0 (0.00%)
*qnrB*	12 (7.36%)	1 (1.43%)	0 (0.00%)
*aac(6′)-Ib-cr*	9 (5.52%)	0 (0.00%)	0 (0.00%)
*ant(3′′)-Ia*	26 (15.95%)	0 (0.00%)	0 (0.00%)
*aph(3′)-IIa*	0 (0.00%)	0 (0.00%)	0 (0.00%)
*tetA*	61 (37.42%)	39 (55.71%)	18 (32.73%)
*tetX*	1 (0.61%)	0 (0.00%)	0 (0.00%)

**Table 5:** Results for minimum free energy prediction of repeat sequences.

Repeat Sequences Number	Repeat Sequence (5′–3′)	Minimum Free Energy Prediction
Sequence 1	GTGTTCCCCGCGCCAGCGGGGATAAACCG	−15.20 kcal/mol
Sequence 2	CGGTTTATCCCCGCTGGCGCGGGGAACAC	−14.20 kcal/mol
Sequence 3	TGTGTTCCCCGCGCCAGCGGGGATAAACCG	−15.20 kcal/mol
Sequence 4	GTGTTCCCCGCGCCAGCGGGGATAAACC	−15.20 kcal/mol
Sequence 5	GTGTTCCCCGCGTCAGCGGGGATAAACCG	−15.20 kcal/mol
Sequence 6	CGGTTTATCCCCGCTGGCGCGGGGAACACG	−14.20 kcal/mol

## Data Availability

The original contributions presented in this study are included in the article/Supplementary Materials. Further inquiries can be directed to the corresponding author.

## Supplementary Materials

The following supporting information can be downloaded at: https://www.mdpi.com/article/10.3390/microorganisms13071683/s1, Table S1: Primers used in this study; Table S2: Isolate strains resistance phenotypic details in this study; Table S3: Statistics on the carriage of antimicrobial resistance genes in isolated *E. coli* strains.

## Abbreviations

The following abbreviations are used in this manuscript:

MDR	multi-drug resistance
CRISPR-Cas	Clustered Regularly Interspaced Short Palindromic Repeats-associated protein
WHO	World Health Organization
ARGs	Antibiotic resistance genes
DR	direct repeats
IUCN	International Union for Conservation of Nature
CLSI	Clinical and Laboratory Standards Institute
r	Pearson correlation coefficient
WGS	whole-genome sequencing
